# Reengineering Workflow for Curation of DICOM Datasets

**DOI:** 10.1007/s10278-018-0097-4

**Published:** 2018-06-15

**Authors:** William Bennett, Kirk Smith, Quasar Jarosz, Tracy Nolan, Walter Bosch

**Affiliations:** 1 Department of Biomedical Informatics, UAMS, 4301 West Markham St, Little Rock, AR 72205 USA; 2 Department of Radiation Oncology, Washington University School of Medicine, St. Louis, MO USA

**Keywords:** Open science, TCIA, De-identification, Curation, DICOM, Image archive, Scalability, Posda

## Abstract

Reusable, publicly available data is a pillar of open science and rapid advancement of cancer imaging research. Sharing data from completed research studies not only saves research dollars required to collect data, but also helps insure that studies are both replicable and reproducible. The Cancer Imaging Archive (TCIA) is a global shared repository for imaging data related to cancer. Insuring the consistency, scientific utility, and anonymity of data stored in TCIA is of utmost importance. As the rate of submission to TCIA has been increasing, both in volume and complexity of DICOM objects stored, the process of curation of collections has become a bottleneck in acquisition of data. In order to increase the rate of curation of image sets, improve the quality of the curation, and better track the provenance of changes made to submitted DICOM image sets, a custom set of tools was developed, using novel methods for the analysis of DICOM data sets. These tools are written in the programming language perl, use the open-source database PostgreSQL, make use of the perl DICOM routines in the open-source package Posda, and incorporate DICOM diagnostic tools from other open-source packages, such as dicom3tools. These tools are referred to as the “Posda Tools.” The Posda Tools are open source and available via git at https://github.com/UAMS-DBMI/PosdaTools. In this paper, we briefly describe the Posda Tools and discuss the novel methods employed by these tools to facilitate rapid analysis of DICOM data, including the following: (1) use a database schema which is more permissive, and differently normalized from traditional DICOM databases; (2) perform integrity checks automatically on a bulk basis; (3) apply revisions to DICOM datasets on an bulk basis, either through a web-based interface or via command line executable perl scripts; (4) all such edits are tracked in a revision tracker and may be rolled back; (5) a UI is provided to inspect the results of such edits, to verify that they are what was intended; (6) identification of DICOM Studies, Series, and SOP instances using “nicknames” which are persistent and have well-defined scope to make expression of reported DICOM errors easier to manage; and (7) rapidly identify potential duplicate DICOM datasets by pixel data is provided; this can be used, e.g., to identify submission subjects which may relate to the same individual, without identifying the individual.

## Introduction/Background

The time required to carry out clinical studies and the high cost of collecting data are motivating the establishment of open science repositories for sharing and re-use of clinical trial datasets. At the same time, concerns regarding protection of patient privacy are strengthening requirements for thorough de-identification of clinical data to avoid disclosure of protected healthcare information. Biomedical investigators typically have limited knowledge of the details of the DICOM standard and often rely on third party vendors of PACS systems and other applications to perform de-identification. Some methods of de-identification, however, may strip information needed to replicate or reproduce a study, and thus, render the data useless for secondary analysis. There is generally no validation step following this de-identification to assure that the data have not been altered in a way that makes it useless, or worse, that would change their interpretation. In addition to altering identification and interpretation of data, such changes may also introduce inconsistencies with respect to the DICOM Patient, Study, and Series data model. This sometimes occurs when DICOM data from multiple sources are combined into a single submission, as is often the case in a large clinical trial.

This paper describes an effort to reengineer the workflow for curation of DICOM images for The Cancer Imaging Archive (TCIA) [10]. TCIA is a service which de-identifies and hosts a large archive of medical images of cancer accessible for public download. TCIA uses the National Biomedical Imaging Archive (NBIA) as a platform to make these images available to the public. As submissions to TCIA have increased in both volume and diversity, several issues in scalability have become evident in the curation process [3] for TCIA:

1. Problems in DICOM conformance may not be detected reliably. Some early collections had separate Frame of Reference Unique Identifiers (UIDs) for each image slice. Thus, the spatial relationship between these DICOM instances, i.e., individual CT slices, was not maintained.
2. DICOM inconsistencies, defined as inconsistent values for shared attributes in a given Entity (Patient, Study, Series) in the DICOM data model, may cause usable data within a DICOM Entity to be “quarantined” at the submitting site or channeled to TCIA “quarantine” directories for manual troubleshooting. Manual repair of these files is a labor-intensive process that requires skilled intervention.
3. The NBIA database normalizes Patient, Study, and Series information, but does not exhaustively verify normalization of imported data. If this information is not consistently normalized across the set of DICOM files belonging to a Patient, Study, or Series, this can result in inconsistencies between DICOM data in the NBIA image database and data in the referenced DICOM file headers.
4. TCIA submissions containing non-image DICOM objects, such as RT Structure Sets, Plans, and Doses, have complex referential relationships. These relationships must be correct for the proper functioning of many types of DICOM workstations.
5. The use of ad hoc scripts to fix problems in DICOM collections makes it difficult to maintain the provenance of submissions. Archive curation requires tracking of all changes to collections to document what was changed, when it was changed, and by whom. Additionally, a validation is required that the expected change was performed and was an accurate repair.
6. Conformant use of DICOM metadata is important to enable interoperable exchange of DICOM information objects. Workstations now make use of the DICOM metadata to determine relationships among images, rather than using long file names and directory hierarchies, as in the past. This is especially important for 3D volumetric datasets.

### Open-Source Toolkits for DICOM Image Manipulations

Software is needed to detect and mitigate errors in DICOM datasets for the curation of data in TCIA. Six open-source tools for display, analysis, and modification of DICOM images were evaluated for this purpose: Dicom3tools [4], CTN [8], DCMTK [5], DVTk [9], DCM4CHE [13], and Posda [11, 2].

While most of these tools provide useful interactive display and editing capabilities for individual datasets, they are not well suited for the high-volume, bulk edits that must be performed on submissions to TCIA. TCIA has been using CTP [6] for performing bulk edits to DICOM images, for de-identification. CTP can be used to perform edits on large batches of DICOM files. In practice, however, acquisition- or PACS-related DICOM errors exhibit a great deal of variability, even within a submission from a single site or scanner. A CTP template script that fixes DICOM errors for one subject in a submission may not be appropriate for all subjects, or some Series types within a collection. It is therefore important to craft an elegant and robust solution that can (a) apply a gross template to address expected changes, and (b) quickly and correctly identify errors, even suggest repairs, to (c) minimize the human time spent in ensuring submitted data conforms to a TCIA standard. The process of isolating errors using existing DICOM dump/analysis tools, formulating CTP scripts to fix the problems, determining the set of files to fix, running the scripts, and confirming that the changes actually fixed the problem is too time-consuming and labor intensive to be economical. For this reason, it was decided to develop tools that would facilitate detection and correction of DICOM errors at scale.

Ultimately, these tools can be integrated into a content management system, such as MIRMAID (Medical Imaging Research Management and Associated Information Database) [7].

### Why Are There So Many DICOM Problems?

The frequency with which errors and inconsistencies are found in collections submitted to TCIA for curation naturally begs the question “Why are there so many DICOM problems in these submissions?” There are three basic reasons:

1. There is frequently no simple correspondence between clinical workflow and data management on one the hand and clinical trial data collection requirements on the other. The process of collecting and preparing clinical data for submission for a multi-institutional clinical trial often require site data managers to maintain information essential to the interpretation of images and related data while anonymizing datasets. As a consequence, the completeness and consistency of datasets in a trial can vary depending on the clinical sites that submitted them.
2. TCIA submissions come from the research community and the clinical community. Often datasets are prepared for publication by researchers using ad hoc methods (scripts) which modify the DICOM. This process may not maintain the DICOM standard expected of The Cancer Imaging Archive.
3. DICOM data that would not be problematic in a single-vendor environment can become problematic when it crosses a vendor boundary, exposing interoperability issues in DICOM implementations. It is therefore critical for TCIA to maintain a knowledge base of private attributes across manufacturers to ensure consistent information is preserved.

## Methods/Materials

The tools used in the curation of DICOM data for TCIA have been implemented using the Posda toolkit [reference]. This toolkit, written in the perl programming language, is available in multiple computing environments. These Posda Tools implement several novel methods for curation of DICOM datasets.

The database schema used by Posda to represent DICOM data is conceptually different from most DICOM databases: Most DICOM databases use a schema which normalizes information according to the DICOM objects, entities, and modules defined in the DICOM data model, i.e., DICOM *as it should be.* By contrast, the Posda DICOM database normalizes according to the encoding structure of the actual DICOM datasets, i.e., DICOM *as it is*. The former is very good for storing conformant DICOM datasets. However, a dataset that does not conform to DICOM must either be rejected (quarantined), or coerced to represent a conformant dataset when imported into the database. The Posda database is capable of storing the DICOM data as it is, and issues of conformance can thus be determined by database queries. No DICOM dataset that can be parsed is discarded for non-conformance. Instead checks on conformance are performed automatically and presented (via a web-based interface) to the user for evaluation and possible correction.

All corrections to DICOM data are performed by supplying an edit specification to a transaction manager and requesting an edit. Correction requests can originate from a web-based curation application or from ad hoc perl scripts written to correct novel problems. Custom perl objects support mapping simple perl analysis/correction scripts over large numbers of files identified by simple nicknames.

All corrections can be tracked as a set of revisions to a dataset, which is identified by the Subject of a submission. Exclusive access to a revision for the duration of an edit is guaranteed through the use of a Transaction Manager. Web-based tools are provided to examine differences between revisions down to the individual element level. Revisions can be easily rolled back to prior versions. Stored with each revision are metadata about the creation of the revision: date/time, user, edit, and user interface operation or script name.

To facilitate recognition by human users, the Posda Tools make use of “nicknames” for DICOM UIDs. Each nickname (about ten characters) is associated with an individual (about 40 characters). Nicknames are persistent and have the scope of a single subject. They provide a user with a simplified way to refer to a file between the Posda Tools UI and an ad hoc perl script or spreadsheet.

The Posda database normalizes Pixel data by computing the MD5 Digest of the pixel data for every image it imports. A benefit of this technique makes it possible to recognize duplicate images despite potentially different UIDs in the database, increasing quantitative information quality of the Archive. These data can be used in strategies to identify subjects which may have accidentally been twice submitted, or refer to the same individual.

## Results

### Fixing Existing Data in TCIA

The first use of the Posda database to diagnose and repair DICOM errors in a large number of datasets in TCIA was to address problems in a large collection which had been in the archive for a number of years. For most, but not all of the image series in this collection, individual “slice” images in one series had each been assigned a Frame of Reference UID (as part of an erroneous attempt at de-identification). Initially, this error did not cause difficulties, as early users of TCIA were just reading the pixel data, assuming that it was a volume, and ignoring all of the spatial information in the DICOM header defining the volume. However, as a larger, more diverse set of users began to download the data, users began to report problems. The data was incompatible with modern, DICOM-based workstations performing volumetric analysis.

At this time, Posda was being used at TCIA for checking (and if necessary, repairing) linkages in RT Objects. The existence of the Posda database was known, but it was not being used.

**Fig. 1:**
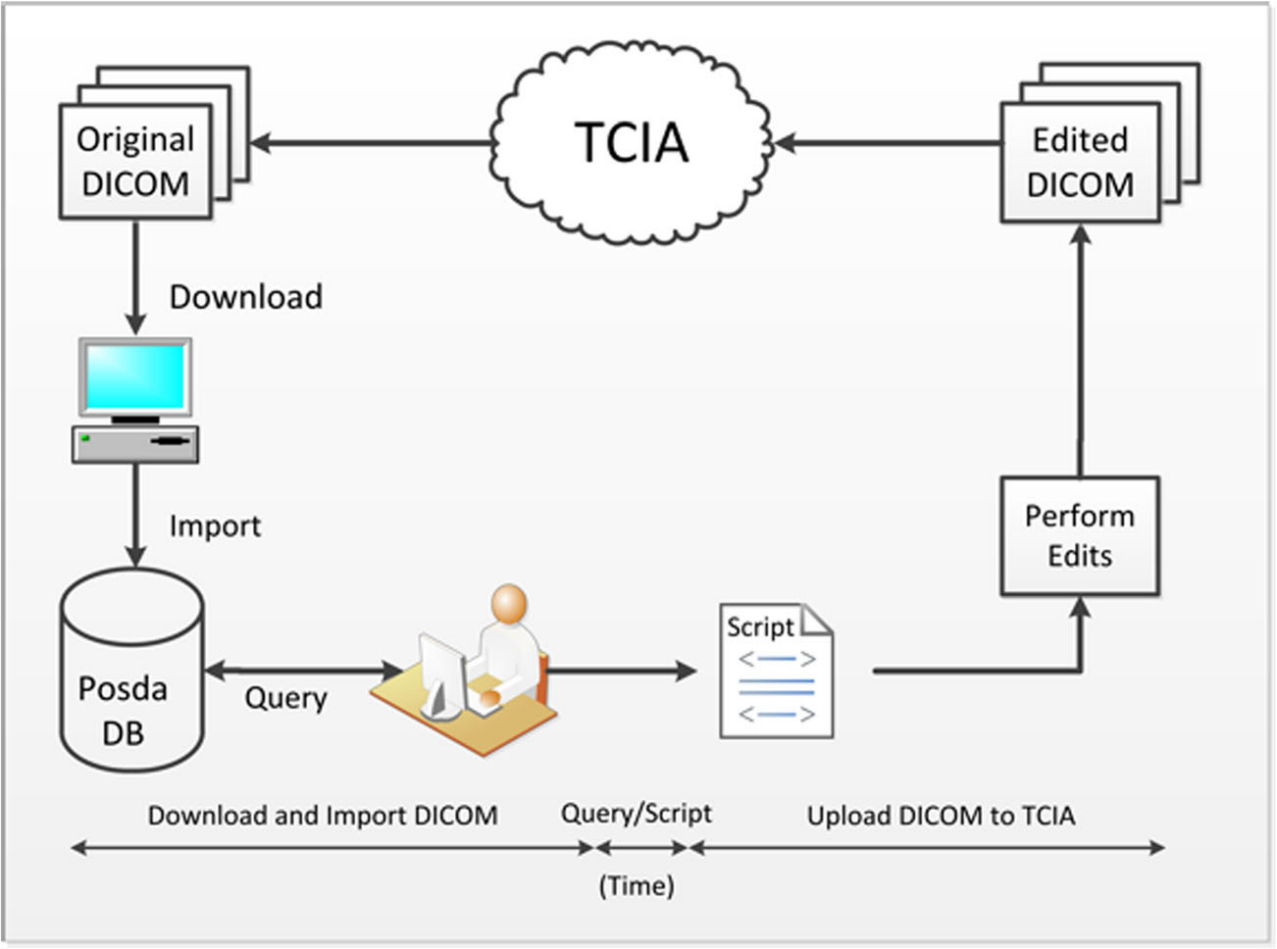
Diagnosis and repair of DICOM errors in TCIA datasets. DICOM data are imported into a Posda database. Queries to this database are used to identify inconsistent Frame of Reference identifiers and generate scripts to correct these inconsistencies. DICOM files are edited before re-loading into TCIA

Owing to the very large number of subjects in the collection to be repaired (over 1000 subjects), and the nature of the tools available for performing DICOM edits, it was proving difficult to script the repair of the datasets. As an alternative, the process shown in Fig. 1 was developed. First, all of the images were imported into the Posda database. This took little scripting time and ran overnight. The database was queried to determine which of the series had inconsistent Frame of Reference UIDs. For each of these series, this information was used to create a command to replace the Frame of Reference UID in each of the remaining files in the series with that of the first file. Finally, the command script was executed to perform edits of the DICOM files and the repaired DICOM files were reloaded into TCIA.

A small perl script was written to perform the database import and query. The output of the perl script, returned in minutes, was a shell script. This shell script was executed overnight to update the Frame of Reference UIDs with consistent values.

The ease with which this problem was resolved using the Posda database highlighted the utility of using a more flexible “intake” database with special characteristics not typically present in a DICOM database. Freed from using normalized Study and Series tables, Posda Tools allowed diagnosis of study and series inconsistencies. This idea seemed so compelling that curation tools based upon the Posda database have become a key part of TCIA curation workflow.

### Diagnosing and Repairing DICOM Inconsistencies

One of the first applications of Posda Tools in the (non-radiotherapy) TCIA workflow was in the diagnosis of series and study inconsistencies in incoming DICOM submissions. Such inconsistencies caused two classes of problems: (1) the NBIA database would reject and quarantine files with Series information that differs from previously imported instances with the same Series Instance, and (2) in other cases, NBIA would accept the data and the database would contain information inconsistent with the DICOM files it would eventually deliver.

**Fig. 2:**
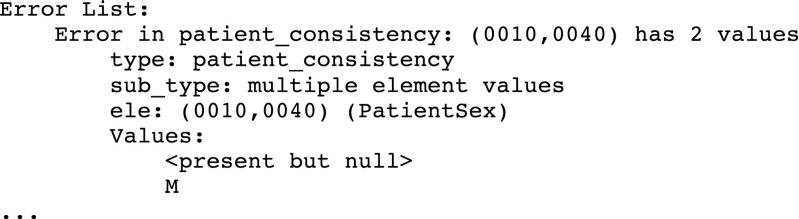
An excerpt from a Consistency Check

In the first implementation of Posda Tools, when DICOM files were received, scripts to diagnose DICOM inconsistences were run automatically. A report of the errors could then be accessed via a web-based application on a per subject basis. An excerpt from such a report is shown in Fig. 2.

Based upon such error descriptions, the user could invoke edits to repair these DICOM inconsistencies: the DICOM header field “patient weight” in some files is missing and in some files is listed. Generating a set of edits to fix these problems requires about 2 min of user time. However, this type of error reporting (by individual subject, with edits to individual subject) created a new problem: these types of edits (which had not been performed before) were consuming a great deal of time. For example, the list above calls out a number of patient and study inconsistencies in a single study of a single subject. When a submission has 250 subjects, this totals over 8 h of work. And this work is particularly tedious and error prone.

Based on the repetitive nature of the work, a method was developed to scan all subjects at once, automatically formulating a set of edits needed to fix the problems found in each selection, and allowing the user to oversee which of the edits would be performed by the software. This new method was found to reduce the curation time to fix errors by over 98%. Specifically, it was used to process a collection which would have taken over 8 h (based on a measured 2 min per subject) in under 9 min [reference second QIN annual meeting poster].

After this feature was implemented in the Posda Tools, however, a submission was received in which a novel type of series inconsistency was detected. For a large number of subjects, there were MR series with hundreds of images, each with a different value for Series Description (0008,103e). The method described above would allow the user to select one of these values to be the Series Description, but this was an unsatisfying solution. While the use of the Series Description to store image-specific annotation represents an abuse of the DICOM data model, this information is nevertheless valuable. This solution was actually equivalent to what was happening in many receiving PACS systems. The PACS would use the series description of the first image received as the description for the entire series, and would ignore the rest. Repairing these series would still require writing ad hoc DICOM editing scripts using manually tabulated data as input. A better solution would be to move the contents of Series Description (perhaps to Image Comment) and replace the contents of Series Description with one that describes the series more accurately. While doing this efficiently is beyond the capabilities of the current Posda Tools, it is the subject of continuing development.

### Applying Other Standard DICOM Diagnostics

In order to provide the highest quality DICOM collections, TCIA strives to use the best available DICOM validation processes. Perhaps the most widely known and used DICOM validator is “dciodvfy,” which is part of the dicom3tools package ([4]–2017). This program examines a single file at a time, and produces a verbose listing of errors and warnings for any DICOM problems found. This tool has been used in the TCIA workflow for some time, but its usage has been limited by manual intervention for the following reasons: (1) the verbose nature of reporting has made the error reports cumbersome to interpret, and (2) the script was run for the first image of every series in an entire collection, but output was sorted by field value absent the source filename of each field in that output as part of the “tag sniffer” program, making it difficult to relate reports in the output of “dciodvfy” to the file (or series) in which the error occurred.

**Fig. 3:**
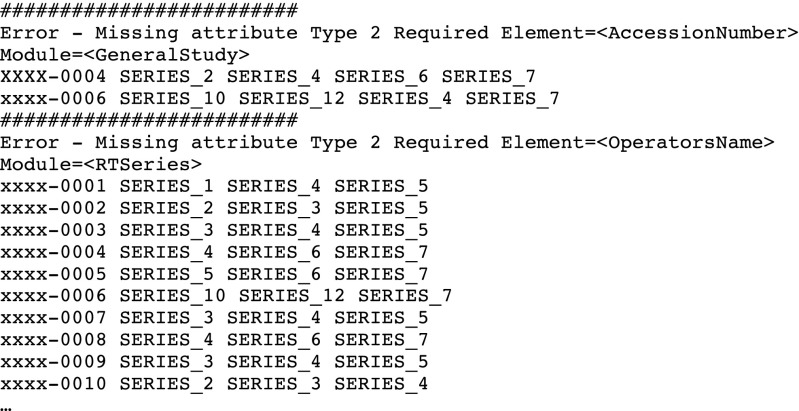
Output from “RunDciodvfy.pl”

As part of the Posda Workflow, a script called “RunDciodvfy.pl” has been developed to run the dciodvfy tool for the first file in every series in a collection, and collect the data based on clusters of reported errors for sets of series. For example, part the output produced by this script is shown in Fig. 3:

In this case, the script has collected errors and the series to which the errors apply and grouped the errors by clusters of series, summarized the errors at the top, and presented the list of series by subject, so that the user can use the Posda UI to explore the cause of the errors. In many cases, it is possible to create a perl script to resolve the errors (such is the case for the two errors above). If this is the case, Posda provides a framework for applying the script to all of the files in the series listed above and performs the edits in a controlled, auditable environment (using the Transaction Manager and constructing revisions). After the edits have been performed, they can be reviewed for correctness in a web-based application and backed out if not correct. The history of the revision (if accepted) will be maintained with a permanent audit trail, maximizing diagnostic and correction effort by TCIA curators.

### Identifying Subjects Who May Represent the Same Individual

The primary purpose of TCIA is to promote reproducible science by providing access to well-curated collections of data, which have been robustly de-identified to protect patient privacy. One of the challenges of using de-identified data is that data may be de-identified more than once. These de-identifications may be done using different methods, by different organizations, using different tools. This can result in data that is ostensibly from two distinct subjects, but actually originated from the same individual. The inclusion of multiple datasets from the same individual can skew the results of analysis based upon this data by inadvertently giving more weight to particular subjects, or by overestimating correlation between supposedly different subject data. Thus, recognizing and eliminating duplicate data is an important goal for curation of collections in TCIA.

The Posda database normalizes its references to pixel data based upon the MD5 Digest of the pixel data. Using a cryptographic hash to recognize equivalent blocks of data is a well-known technique for identifying duplicate blocks of arbitrary data [12]. The MD5 digests stored in the Posda database allow for easy recognition of files with the same pixel data. Patterns of duplicate pixel data can then be used to detect the presence of duplicated patient data within a collection.

An analysis of the patterns of duplicate pixel digests leads to the following observations:

1. If a series is duplicated, it will mean a large number of pairs of duplicate pixel data (one for each file in the series).
2. A query that produces a large number of distinct pixel digests, each with a small of number of duplicate instances indicates a possible duplicated series. For example, if there are 195 digests each of which occur in exactly three files, then it is possible that a single series with 195 images was duplicated twice (see Fig. 5).
3. Certain “distinguished” pixel digests result from various sizes of blank images. These digests will tend to have a large number of files with duplicate pixels in a large collection and may be ignored (see Fig. 5).
4. Another category of “distinguished pixel digests” can arise from test images (SMPTE patterns, electronic phantoms, etc). These duplicates may also be ignored.
5. A small number of duplications of pixel digests may result from the copying of “key images” as scout or locator image for a new series of the same patient.

**Fig. 4:**
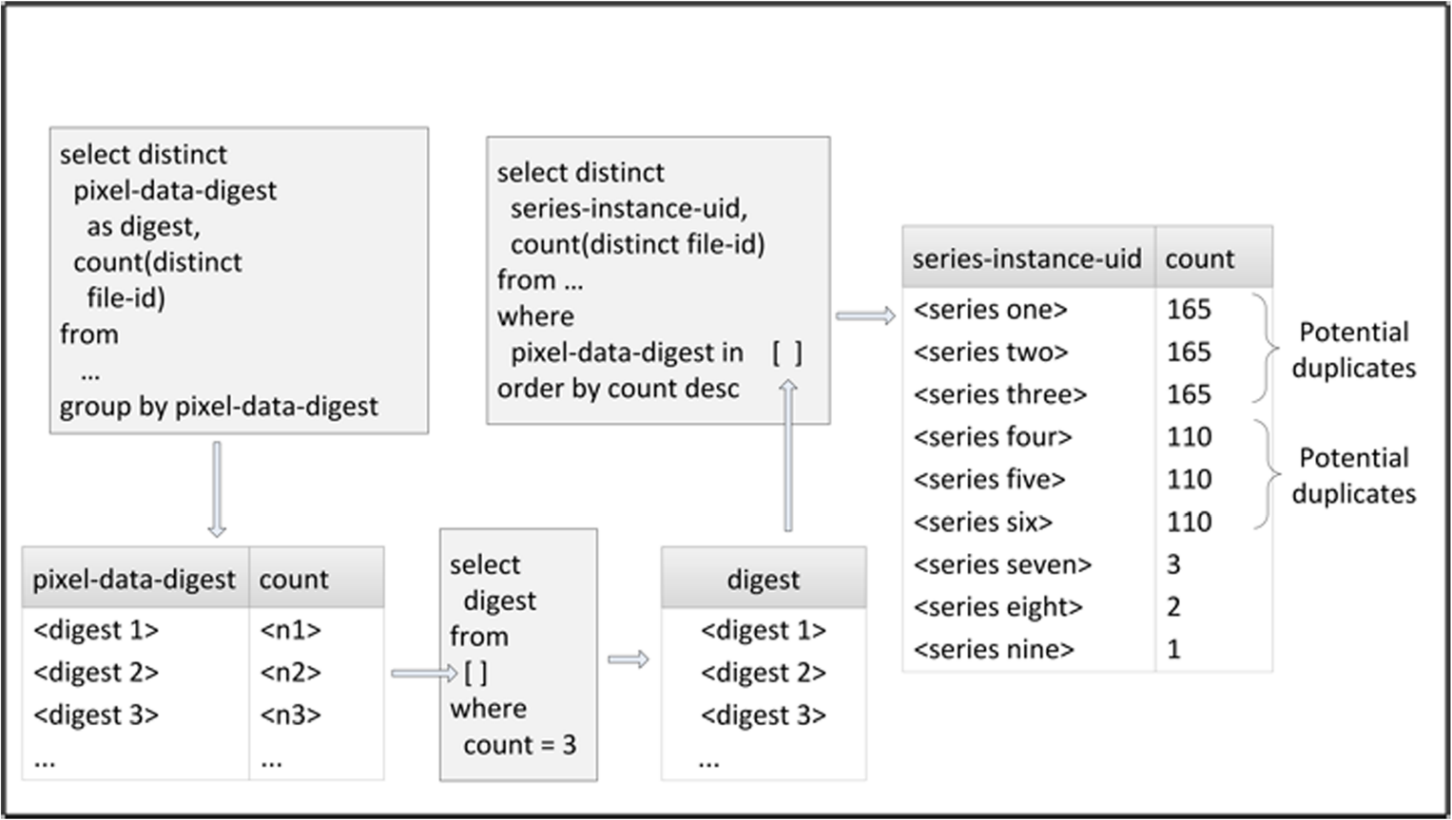
Process for finding potential duplicate series based upon finding series which have the same number of files with duplicate pixel data

Figure 4 below shows a process for finding potential duplicate series based upon finding series which have the same number of files with duplicate pixel data.

**Fig. 5:**
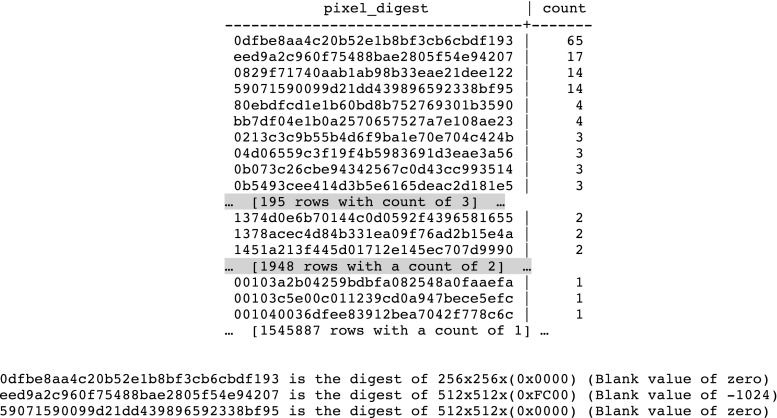
Distinguished digests

Figure 5 below shows the result of a query for unique pixel digests, with a count of files for each digest, followed by a list of a few commonly observed pixel data digests for blank images.

**Fig. 6:**
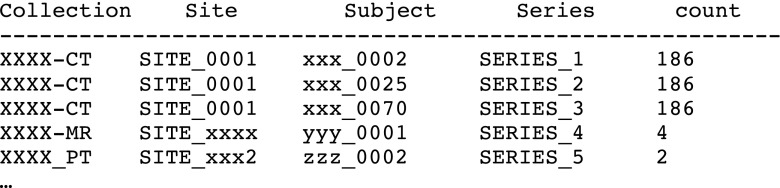
List used to identify potential duplicate series

The 195 rows of digests which are duplicated three times each suggests that there may be a series which has been duplicated twice. The results of a query that groups the twice duplicated digests by collection, site, and series containing the digests in question yields the following results (anonymized collection, site, subject, and series_id) is shown in Fig. 6.

**Fig. 7:**
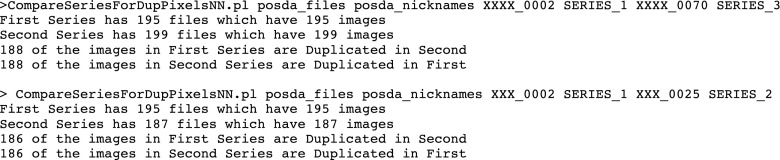
Determining actual duplicate series and extent of duplication

This example identifies three series as potential duplicates of one another. To check and see if they actually are duplicates, there is another perl script that compares two series for duplicates. The results of running this script are shown in Fig. 7.

**Fig. 8:**
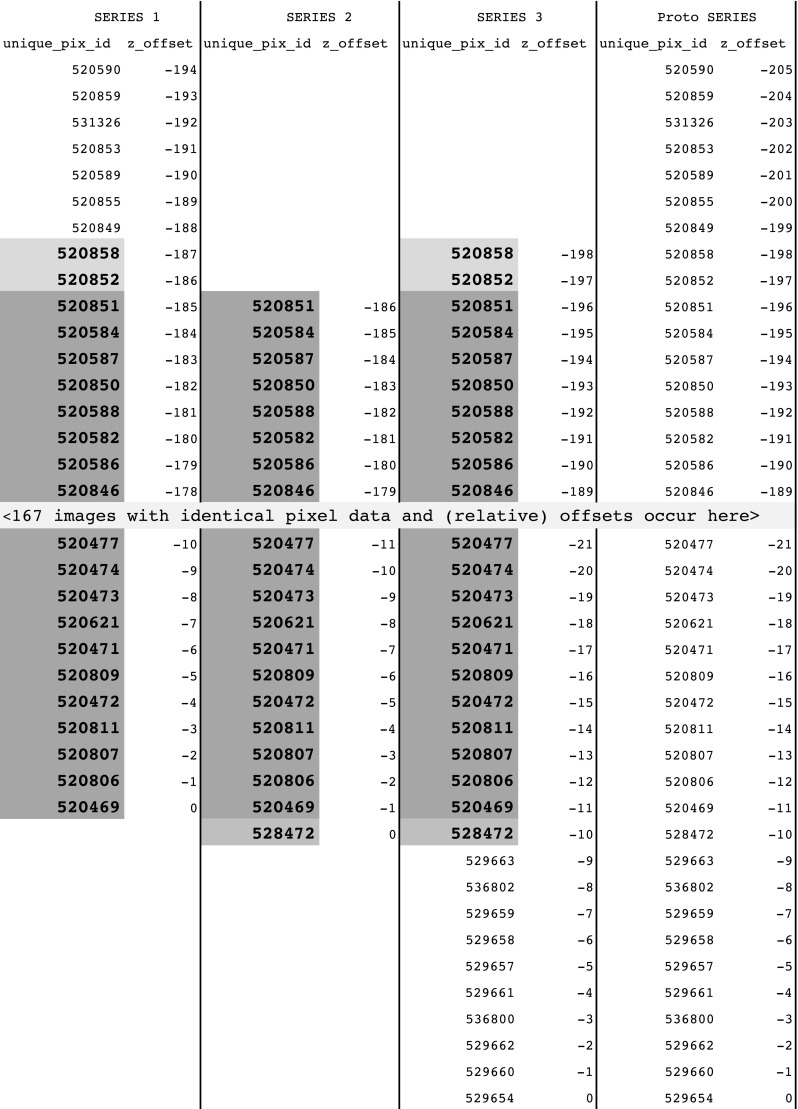
Columns for each of the Series 1–3 and “Proto” Series

Figure 8 illustrates the images actually found in the three series in question, and their relationship to one another. By examining this data, we can infer the existence of a “proto” series with 206 images, with z-offsets ranging from − 205 to 0. Series 1 includes the first 195 images and renumbers the offsets starting at − 194. Series 2 skips the first 9 images, includes the next 187 images, and renumbers the offsets starting at − 186. Series 3 skips the first 7 images, includes the remaining 199 images, and renumbers the offsets starting at − 198 (actually no renumbering required, in the original, i.e., proto series the 8th image already had an offset of − 198). So three of the original images are duplicated once (marked in gray in Fig. 8) and 186 of the images are duplicated twice (marked in darker gray in Fig. 8). The 186 images duplicated twice attract attention in the query in Fig. 6.

A similar analysis was conducted on the 1948 single duplications of pixel data shown in Fig. 5 (rows with a count of 2). This analysis is not detailed here, but it revealed four instances of duplication of patient data. In one of these instances, it was a patient who was a participant in two different studies, and imaging information for the patient was collected for both studies. In the other three, the duplicate data was included erroneously. In two of the cases, inclusion of the duplicates would have seriously compromised the usability of the data. In these cases, there was identical data in the “training” and “test” sets which would compromise validity of research results when using this collection.

## Discussion

The applicability of Posda-based tools has been demonstrated in three important areas in the curation of DICOM images collections: (1) detection and mitigation of attribute value inconsistencies in DICOM instances at the Subject, Study, and Series level; (2) detection and mitigation of DICOM SOP Class Conformance issues; and (3) detection and elimination of duplications of DICOM data at the Subject level.

The Posda Tools described in this report can detect DICOM inconsistencies at various levels. For some collections, they have been shown to be effective at efficiently resolving such inconsistencies in their current form. For other collections, however, methods other than those implemented in this tool set are required to resolve inconsistencies. The interfaces needed to implement these methods have yet to be created. It is not clear whether a single toolset can be created to address all such problems: developing new human interfaces for each new type of inconsistency seems to be an ever-escalating (and relatively expensive) effort. A common source of inconsistencies results when implementers do not find a place to store information within the DICOM standard and resort to encoding data in ways that do not conform to the DICOM Data model. Such is the case when implementers choose to store image information in series description. While many clinical PACS systems may be immune to such DICOM inconsistencies, curators of large collections have a responsibility to move the community towards more consistently structured (and therefore, more easily queried) collections. Therefore, work in this area needs to continue, and more research into different types of analysis methods and user interfaces is needed.

It is not a new idea to use a database which is not normalized to the DICOM data model to store DICOM data. [1]. These techniques are mainly employed to allow Image Storage systems to allow a broader set of document-style queries to be performed. This contrasts with the Posda database, the purpose of which is to allow specialized queries to detect ways in which the data does not conform to the DICOM data model, so that the individual files can be modified to conform, allowing the data to be successfully imported into traditional PACS systems which enforce this data model.

The tool “dcentvfy” tool, which is part of the dicom3tools package [4], is well known for its ability to detect a wide variety of DICOM inconsistencies. It should be noted that finding DICOM inconsistencies is not the problem, it is determining and applying the appropriate fix for the inconsistences in a scalable fashion.

In terms of detecting DICOM conformance issues, the “dciodvfy” utility does a remarkably good job, but requires a “wrapper” program to collect and quantify its output. The Posda Tools perform reasonably well as “wrapper” scripts, and in many cases can be used to correct problems in DICOM conformance. However, the step between detection and resolution is still difficult to automate and may require consultation with the submitter of the data to confirm interpretation of inconsistencies and provide additional information needed to resolve the differences appropriately.

Detecting duplicate subjects on the other hand yields a vast qualitative improvement in the curation of DICOM image sets. Revealing previously unknown duplicates has applications in many areas. These include (a) improving interpretation of research by eliminating spurious correlations, (b) insuring strict separation of data between training and testing data sets for challenges, (c) reducing the amount of data stored by identifying (for example) CT series which have been re-exported by segmentation systems, and (d) detecting fraudulent submission of data.

## Conclusions

The key idea behind the Posda Tools is that there should be a different kind of DICOM database (a “curation” database) for curation of DICOM Image sets. This database is different from other DICOM databases (PACS databases), in that its schema is designed to record “DICOM as it is” rather than “DICOM as it should be”. Some tables (such as DICOM series) are de-normalized to allow inconsistencies in the DICOM specified hierarchy to be represented in the database. Other tables (such as “Image” table) are normalized around different characteristics (such as pixel data digest) to allow recognition of duplicates.

Use of a curation database has been shown to improve the ability to detect DICOM inconsistencies, find DICOM errors, and detect duplications of DICOM data in large collections of DICOM images. It also facilitates mitigation of these detected problems, and for certain types of DICOM inconsistencies supports efficient resolution of DICOM inconsistencies. However, for certain other types of DICOM inconsistencies, tools for resolution efficiently still remain to be written, and the novelty of inconsistency type has not yet abated. To avoid a continuing need for new user interfaces, a novel paradigm for user interaction may be required.

## References

[1] Bastião Silva L, Beroud L, Costa C, Oliveira J: Medical imaging archiving: a comparison between several NoSQL solutions. Biomedical and Health Informatics (BHI), 2014 IEEE-EMBS International Conference On. 2014. pp 65–68. 10.1109/BHI.2014.6864305.

[2] Bennett W (2010). SU-GG-T-262: Open-source tool for assessing variability in DICOM data. Med Phys.

[3] Clark K (2013). The Cancer Imaging Archive (TCIA): maintaining and operating a public information repository. J Digit Imaging.

[4] Clunie DA: Dicom3tools Software. 1995–2017, 11/22/2009. Retrieved 5/22/2017, 2017, from http://www.dclunie.com/dicom3tools.html

[5] Eichelberg M, et al.: Ten years of medical imaging standardization and prototypical implementation: the DICOM standard and the OFFIS DICOM toolkit (DCMTK). Medical Imaging 2004, International Society for Optics and Photonics. 2004

[6] Freymann JB (2012). Image data sharing for biomedical research—meeting HIPAA requirements for de-identification. J Digit Imaging.

[7] Korfiatis PD (2015). MIRMAID: A content management system for medical image analysis research. Radiographics.

[8] Moore SM, et al: DICOM shareware: a public implementation of the DICOM standard. SPIE 2165, Medical Imaging 1994: PACS Design and Evaluation, Newport Beach, CA, International Society for Optics and Photonics. 1994

[9] Potter G (2007). Mastering DICOM with DVTk. J Digit Imaging.

[10] Prior FW, et al: TCIA: an information resource to enable open science. Engineering in Medicine and Biology Society (EMBC), 2013 35th Annual International Conference of the IEEE, Osaka, Japan, IEEE. 2013 10.1109/EMBC.2013.6609742 PMC4257783 24109929

[11] Rosenstein BS (2016). How will big data improve clinical and basic research in radiation therapy?. Int J Radiat Oncol Biol Phys.

[12] Tridgell A, Mackerras P: The rsync algorithm. 1996

[13] Zeilinger G, et al: The dcm4che project homepage. tech.rep.dcm4che.org. 2010

